# Wearable Inertial Sensor Analysis of Turning Performance Reveals Motor Reserve Effects in Drug-Naïve Parkinson’s Disease

**DOI:** 10.3390/s26092594

**Published:** 2026-04-22

**Authors:** Andrea Rizzardi, Cinzia Zatti, Alice Galli, Mohsen Fallahi, Sofia Bonomelli, Nicolò Agostini, Klaudia Eshja, Martina Ogliani, Veronica Pucci, Massimo Nucci, Sara Mondini, Clint Hansen, Robbin Romijnders, Walter Maetzler, Alessandro Padovani, Andrea Pilotto

**Affiliations:** 1 Neurology Unit, Department of Clinical and Experimental Sciences, University of Brescia, 25123 Brescia, Italy; c.zatti002@unibs.it (C.Z.); alice.galli@unibs.it (A.G.); mohsen.fallahi@unibs.it (M.F.); sofia.bonomelli@unibs.it (S.B.); k.eshja@unibs.it (K.E.); alessandro.padovani@unibs.it (A.P.); andrea.pilotto@unibs.it (A.P.); 2 Laboratory of Digital Neurology and Biosensors, University of Brescia, 25123 Brescia, Italy; 3 Neurology Unit, Department of Continuity of Care and Frailty, ASST Spedali Civili Brescia Hospital, 25123 Brescia, Italy; 4 Department of Philosophy, Sociology, Education and Applied Psychology (FISPPA), University of Padova, 35122 Padova, Italy; veronica.pucci@phd.unipd.it; 5 Human Inspired Technology Research Centre, University of Padova, 35122 Padova, Italy; massimo.nucci@unipd.it (M.N.); sara.mondini@unipd.it (S.M.); 6 Department of General Psychology, University of Padova, 35122 Padova, Italy; 7 Department of Neurology, University Hospital Schleswig-Holstein, 24105 Kiel, Germany; c.hansen@neurologie.uni-kiel.de (C.H.); r.romijnders@neurologie.uni-kiel.de (R.R.); w.maetzler@neurologie.uni-kiel.de (W.M.); 8 University Medical Center Schleswig-Holstein, Campus Kiel, 24105 Kiel, Germany; 9 Brain Health Center, University of Brescia, 25123 Brescia, Italy; 10 Division of Clinical Geriatrics, Center for Alzheimer Research, Department of Neurobiology, Care Sciences and Society (NVS), Karolinska Institute, 171 77 Stockholm, Sweden

**Keywords:** motor reserve, Parkinson’s disease, de novo, turning, mobile health technology

## Abstract

**Introduction**: Motor reserve (MR) has been hypothesized as a protective factor against age-related and pathological motor decline, potentially enhancing quality of life. This study aimed to investigate the influence of MR on motor performance, assessed via mobile health technology (MHT), in drug-naïve Parkinson’s disease (PD) patients. **Methods**: Consecutive drug-naïve PD patients and age-matched healthy controls (HC) underwent cognitive and motor assessments. Turning MHT parameters were extracted from the Timed Up and Go test (TUG) performed at self-selected and fast speeds. Participants were categorized into high- or low-MR groups based on the Motor Reserve Index questionnaire (MRIq). **Results**: Forty-five PD patients and forty healthy controls (HC) were enrolled. PD patients showed longer TUG durations and altered performance compared to HC. No differences were found between high and low motor reserve (MR) groups in demographics or clinical severity. However, high-MR patients exhibited shorter turn duration and higher angular velocities at both self-selected (*p* < 0.005) and fast speeds (*p* < 0.05). MR subdomains related to physical and care activities correlated with MHT turning metrics, unlike housework and leisure domains. **Conclusions**: the findings highlighted the relevance of MR on motor performances assessed by MHT in drug naïve PD, independently from motor severity.

## 1. Introduction

Motor reserve (MR) is an emerging concept in neurodegenerative research that refers to the brain’s ability to compensate for pathological damage in motor networks, thereby maintaining motor performance despite structural or functional impairments. Analogous to the well-established concept of cognitive reserve in Alzheimer’s disease and other dementias [1], MR could explain why interindividual variability in motor function is not solely the degree of neuropathology. Instead, it is influenced by various protective or compensatory mechanisms, which may include enhanced synaptic efficiency, alternative motor pathway recruitment, or neuroplastic adaptation [2,3]. Factors that may contribute to MR include genetic predispositions, lifelong physical activity, engagement in motor learning activities and overall brain health. Understanding MR in the context of Parkinson’s disease (PD) may have important clinical implications, as it could inform prognostic models, identify individuals at risk of rapid decline, and guide personalized therapeutic interventions [4]. The Timed Up and Go (TUG) test is a well-established, standardized clinical assessment used to evaluate basic functional mobility in individuals with PD and the general population [5]. It provides a global measure of mobility that captures dynamic balance, gait speed, and lower limb strength. In the context of PD, TUG performance has been linked to disease severity, fall risk, and functional independence [6]. Traditional Timed Up and Go (TUG) assessments rely solely on total time to completion, which may fail to detect subtle motor impairments or group differences, especially in specific components of movements. To overcome this limitation, instrumented TUG protocols have been developed by incorporating wearable inertial measurement units (IMUs).

These metrics provide greater sensitivity to subtle motor alterations, enabling a more detailed and comprehensive assessment of motor function, particularly during turning [7].

Recently, attempts have been made to operationalize the concept of motor reserve through structured instruments that quantify lifetime engagement in motor-enriching activities. The Motor Reserve Index questionnaire (MRIq) is a recently developed tool designed to capture cumulative exposure to motor behaviors across different domains of daily life, including household activities, walking, leisure activities, physical exercise, care activities, and work-related motor engagement [7]. These domains reflect long-term engagement in motor experiences that may contribute to reserve-related mechanisms. Although originally proposed in the context of aging and cognitive variability, MRIq has been suggested as a behavioral proxy of motor enrichment potentially relevant for the study of motor reserve in neurodegenerative conditions.

The aim of this study is to explore the role of motor reserve in Parkinson’s disease by analyzing digital TUG performance. Specifically, we examine the impact of a novel motor reserve index on MHT-derived parameters, independently of clinical motor severity

## 2. Methods

### 2.1. Participants and Clinical Assessment

This investigation followed a prospective observational design conducted at a single center.

Individuals attending the Movement Disorders outpatient clinic at the Neurology Unit of the University of Brescia (Italy) between January 2023 and March 2024 were consecutively considered for inclusion. The study protocol was approved by the local Ethics Committee (DMA study, NP 1471; latest amendment 22 April 2022), and written informed consent was obtained from all participants prior to enrollment.

The cohort comprised drug-naïve patients with a clinical diagnosis of Parkinson’s disease, together with age-matched healthy controls. Exclusion criteria were defined as follows: (i) clinical features suggestive of atypical parkinsonism; (ii) presence of cognitive impairment; (iii) neurological or systemic conditions potentially affecting gait; (iv) structural abnormalities detected on brain magnetic resonance imaging; (v) requirement for walking aids; and (vi) evidence of preserved dopaminergic function at nigrostriatal imaging.

Additional exclusion criteria included age below 18 years, history of major psychiatric disorders (including mood or psychotic conditions), impulse control disorders, unstable or acute medical conditions, and pharmacological or clinical factors potentially influencing cognitive or affective status. Healthy controls were recruited among caregivers and volunteers.

Each participant completed a structured neurological evaluation including the Movement Disorder Society–Unified Parkinson’s Disease Rating Scale (MDS-UPDRS) [8], Hoehn and Yahr staging [9], and cognitive screening using the Montreal Cognitive Assessment (MoCA) [10].

Motor reserve was operationalized using the Motor Reserve Index questionnaire (MRIq) [7] (Supplementary File S1), which captures lifetime engagement in motor-related activities across multiple domains. As this instrument has been only recently introduced and lacks validated thresholds for Parkinson’s disease populations, participants were stratified using a median split approach. This exploratory strategy is consistent with previous reserve-based research relying on behavioral proxies to categorize individuals according to reserve levels [1,2,3,4]. Based on the median value, patients were classified into high motor reserve (High MR) and low motor reserve (Low MR) groups.

Healthy controls were included to provide a reference framework for digital mobility parameters and to assess the sensitivity of instrumented measures in detecting Parkinsonian motor alterations.

### 2.2. Mobile Health Technology Instrumented Timed Up and Go Assessment and Gait Analysis

Functional mobility was examined through the Timed Up and Go (TUG) test, a standardized task incorporating transitions between sitting and standing, walking, turning, and sitting. Each participant performed the test under two conditions (self-selected and fast speed) and in both clockwise and counterclockwise directions, following established procedures [11].

Kinematic signals were collected through a wearable inertial measurement unit (Rehagait^®^, Hasomed, Germany) equipped with accelerometers and gyroscopes. The sensor was positioned at the level of the fifth lumbar vertebra (L5), approximating the body’s center of mass.

Raw signals were subsequently exported and processed offline using custom-developed scripts in MATLAB (R2021a). Individual trials were identified through time-stamp segmentation. Turning phases and associated features were extracted using a validated algorithm described by Pham et al. [12], which includes three main steps: estimation of sensor orientation based on a six-degree-of-freedom (6DOF) model, identification of turning segments, and computation of turning-related metrics.

The main outcome measures were turning duration, mean angular velocity, and peak angular velocity, selected based on previous evidence supporting their sensitivity to early motor alterations in Parkinson’s disease [13].

Variables with more than 5% missing values were excluded from the analyses. Outliers were defined as values exceeding ±3 standard deviations within each group and were removed prior to statistical evaluation.

To quantify performance differences across conditions, percentage variation between self-selected and fast speed was calculated for angular velocity measures using the following formula: (fast − self-selected)/self-selected.

### 2.3. Statistical Analysis

All statistical procedures were performed using IBM SPSS Statistics version 26.

Normality of continuous variables was evaluated through the Shapiro–Wilk test. Depending on data distribution, between-group comparisons for demographic and clinical variables were conducted using Mann–Whitney U tests or chi-square tests, as appropriate.

Differences in turning parameters between high and low motor reserve groups were analyzed using analysis of covariance (ANCOVA), with adjustment for age, sex, and MDS-UPDRS part III scores.

Associations between MRIq scores and digital mobility outcomes were examined using partial correlation analyses controlling for age and sex.

Considering the exploratory nature of the study, statistical significance was set at *p* < 0.05 without applying corrections for multiple comparisons.

In accordance with previous literature [13], mean values derived from clockwise and counterclockwise turns were used. No formal sample size calculation was performed due to the observational design of the study.

## 3. Results

Forty-five PD patients and forty age-matched controls entered the study. All subjects completed all MHT procedures without discomfort.

The overall duration of the TUG tests was longer in PD compared to controls, both at self-selected and fast speed. The HC subjects showed higher angular and peak angular velocity and lower duration of turns (*p* < 0.01) (Supplementary Table S1).

Participants were divided into two groups according to the median value of the MRI Total Score in subjects with high and low motor reserve. High vs. low-MR PD groups were comparable for demographics but not in height, motor, and cognitive severity and clinical variables (Table 1). Additionally, MRI total score did not correlate with motor severity assessed by MDS-UPDRS-III. Of note, cognitive reserve did not correlate with motor reserve and did not correlate with any motor performance.

High-MR PD showed lower duration of turns and higher angular velocity and peak angular velocity both at self-selected speed (*p* < 0.005) and at fast speed (*p* < 0.05). No differences were found in the rates of change between self-selected and fast conditions. (Table 2).

**Figure 1:**
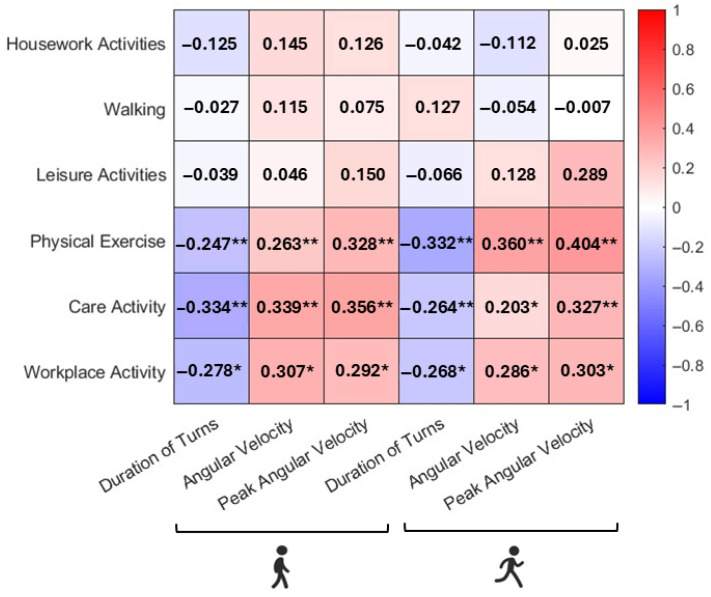
Correlations between digital parameters and MRI subitems. Partial correlation adjusted for age and sex during self-selected pace TUG and fast TUG. * *p* < 0.05, ** *p* < 0.01.

Partial correlations adjusted for age and sex revealed a significant relationship between digital parameters both in self-selected and fast pace and MRIq (*p* < 0.05). However, the strength of these correlations was generally low, indicating modest effect sizes. Subitems analysis revealed that section IV, related to physical activities, and section V, related to care activities, were strongly correlated with durations of turns, angular velocity, and peak velocity (all *p* < 0.01), whereas section I and section III, related to housework and leisure activities, did not show any correlations (Figure 1).

## 4. Discussion

Our results suggest that motor reserve, operationalized through the Motor Reserve Index questionnaire, is associated with differences in turning performance in drug-naïve PD patients. The results were supported by a standardized and innovative approach to MR assessment [7], combined with the digital assessment of mobility using a well-established performance measure such as the instrumented Timed Up and Go. Importantly, these effects appeared to be independent of motor severity and cognitive reserve. These results support the hypothesis that MR acts as a compensatory mechanism in the early stages of PD and highlight the value of digital tools in capturing subtle motor variations not detected by conventional clinical rating scales.

The notion of reserve, initially developed in the field of cognitive neuroscience to explain variability in cognitive decline in Alzheimer’s disease, has more recently been extended to motor domains. In parallel, non-invasive approaches for Parkinson’s disease assessment have increasingly relied on wearable sensors, digital biomarkers, and data-driven analytical frameworks, including machine learning techniques, often focusing on classification or prediction of motor impairment. While these approaches have shown promising results, they frequently rely on complex analytical models that may limit direct clinical interpretability [14,15].

Within this context, the present study adopts a complementary and clinically oriented perspective, focusing on the association between motor reserve and specific mobility features derived from a standardized and widely used test such as the Timed Up and Go. Rather than classifying disease status, our approach aims to investigate how reserve-related mechanisms may modulate motor performance, providing insight into interindividual variability that is not captured by classification frameworks.

Importantly, this approach extends current digital assessments by linking quantitative motor features to an underlying reserve-based mechanism, thereby providing a pathophysiological interpretation of interindividual variability that is not addressed by purely data-driven classification models. Motor reserve refers to the capacity of the motor system to withstand neuropathological changes while maintaining performance. In the context of PD, where the degree of nigrostriatal dopaminergic loss does not always directly correspond to clinical symptoms or disability levels, MR may account for the observed heterogeneity in motor presentation and progression rates. Our findings align with this framework by showing that high-MR PD patients consistently outperformed low-MR patients in turning metrics at both self-selected and fast walking speeds. Importantly, this advantage remained significant after adjusting for age and sex and was not associated with differences in cognitive reserve or global motor severity scores. The lack of correlation between MR and MDS-UPDRS-III further supports the idea that reserve-related functional advantages are not necessarily reflected in motor severity assessed by standard clinical scales.

Specifically, by combining wearable-based turning metrics with a behavioral proxy of motor reserve (MRIq), our approach aims to bridge the gap between digital quantification and pathophysiological interpretation.

Of note, we observed a similar, less pronounced effect at fast speed, likely due to a greater variation in duration changes between self-selected and fast speeds in low MR PD patients compared to high MR patients. We hypothesize that this could be due to the activation of cortical circuits in low MR patients during motor tasks, compensating for early subcortical impairments, as recently demonstrated in prodromal PD [16]. In contrast, subcortical networks responsible for routine mobility may be affected later in high MR patients, while cortical networks are more engaged in handling complex or demanding tasks. Moreover, the analysis of MRIq subdomains revealed that the MR effect was primarily driven by domains related to physical and care activities, whereas leisure and housework domains did not show significant associations with motor parameters. This finding supports the hypothesis that active and physically demanding experiences may play a more direct role in building or maintaining motor reserve than more passive or sedentary tasks. Notably, although statistically significant, the observed correlations were of low magnitude and should therefore be interpreted with caution.

These findings may have several relevant clinical implications. First, they suggest that MR is a meaningful construct in PD that can influence early motor performances. Identifying patients with low MR could support earlier and more targeted interventions, including physical activity programs aimed at enhancing motor plasticity and compensation. Second, our results highlight the potential of integrating digital biomarkers into clinical assessments to improve the sensitivity and precision of motor evaluation in PD. While the MDS-UPDRS remains the gold standard for clinical staging, it may not adequately reflect subtle differences in functional performance, particularly in the early stages of the disease. Digital motor assessments offer a scalable and objective way to capture real-world motor behavior and complement traditional assessments [17,18].

Several limitations should be acknowledged. The study is based on a single-center cohort of drug-naïve PD patients, which allows for a controlled assessment in early disease stages but may limit the generalizability of the findings to broader PD populations. The cross-sectional design precludes conclusions about causality or longitudinal effects of MR on disease progression. Although major confounders were controlled for, additional factors such as cardiovascular fitness may contribute to MR and were not assessed. Furthermore, we did not include an unsupervised assessment, as the features related to bradykinesia are expected to be more evident in the home-setting compared to supervised evaluation in the clinic [17]. Finally, while our digital analysis focused on turning, other motor domains such as postural transitions, gait variability, or dual-task performance may also be influenced by MR and merit further investigation using specific on-going tasks [11].

Future studies combining imaging biomarkers with digital mobility measures may help clarify whether motor reserve moderates the relationship between nigrostriatal degeneration and motor performance.

In conclusion, our findings support the hypothesis that motor reserve may contribute to variability in motor performance in early PD. Using digital mobility assessments, we show that high MR is associated with more efficient turning performance, independently of clinical severity. These findings highlight the importance of considering reserve mechanisms in both research and clinical practice, and support the integration of wearable technologies in routine PD assessment to improve patient stratification and personalized management.

## Figures and Tables

**Table 1:** Clinical characteristics of patients.

	**Low MR**	**High MR**	***p*** **-Value**
Participants [n]	27	18	
Age [years]	68.8 ± 7.7	69.5 ± 7.5	0.571 ^b^
Sex [% male]	47.6	28.6	0.204 ^a^
Disease duration [years]	2.8 ± 1.4	3.1 ± 1.7	0.732 ^b^
MDS-UPDRS III, total score	18.1 ± 7.6	16.7 ± 8.6	0.290 ^b^
H&Y stage	2.1 ± 1.1	2.0 ± 1.3	0.731 ^b^
MoCA, total score	27.5 ± 4.6	28.7 ± 1.1	0.467 ^b^
Education [years]	8.1 ± 2.7	9.3 ± 3.4	0.326 ^b^
Height [m]	1.69 ± 0.01	1.64 ± 0.01	0.035 ^b^
BMI	24.9 ± 3.8	24.5 ± 3.6	0.772 ^b^
CRI Education	104.7 ± 13.8	102.5 ± 11.2	0.960 ^a^
CRI Work	98.9 ± 17.8	99.8 ± 17.6	0.514 ^a^
CRI Leisure	105.1 ± 16.0	107.8 ± 19.5	0.151 ^a^
CRI Total Score	103.6 ± 17.0	104.3 ± 12.9	0.221 ^a^
MRI Home Activities	14.0 ± 11.5	28.1 ± 18.2	0.017 ^a^
MRI Walking	7.9 ± 6.4	21.7 ± 20.2	0.079 ^a^
MRI Leisure Activities	12.4 ± 7.2	23.4 ± 19.9	0.005 ^a^
MRI Physical Exercise	7.0 ± 6.5	16.4 ± 13.0	0.022 ^a^
MRI Care Activities	14.1 ± 4.2	22.5 ± 10.9	0.022 ^a^
MRI Workplace Activities	19.1 ± 9.9	22.8 ± 12.3	0.650 ^a^
MRI Total Score (%)	12.1 ± 1.8	21.7 ± 9.0	0.000 ^a^

^a^ Chi-square test; ^b^ U Mann-Whitney test.

**Table 2:** Turning characteristics of the patients.

Turning Parameters	Low MR	High MR	*p*-Value
	**TUG Self Selected Speed**		
TUG Duration [s]	17.8 ± 7.6	16.1 ± 4.7	0.220 ^a^
Postural Transition Phase [s]	1.7 ± 0.3	1.6 ± 0.6	0.756 ^a^
Walking Phase [s]	12.4 ± 6.6	11.8 ± 4.9	0.591 ^a^
Duration of turns [s]	3.4 ± 0.7	2.7 ± 0.7	0.003 ^a^
Mean Angular Velocity [°/s]	53.7 ± 10.8	67.8 ± 13.7	0.001 ^a^
Peak Angular Velocity [°/s]	119.6 ± 20.1	152.6 ± 31.4	0.000 ^a^
	**TUG Fast Speed**		
TUG Duration [s]	14.0 ± 5.9	12.7 ± 3.6	0.231 ^a^
Postural Transition Phase [s]	1.3 ± 0.2	1.2 ± 0.6	0.676 ^a^
Walking Phase [s]	9.5 ± 4.8	8.7 ± 3.9	0.579 ^a^
% Variation Tug Duration	−27.9 ± 11.0	−27.1 ± 9.8	0.748 ^a^
Duration of turns [s]	2.8 ± 0.8	2.3 ± 0.5	0.034 ^a^
% Variation Duration of Turns	−20.9 ± 19.3	−16.0 ± 14.2	0.475 ^a^
Mean Angular Velocity [°/s]	68.2 ± 16.7	77.0 ± 14.6	0.024 ^a^
% Variation Mean Angular Velocity	17.9 ± 9.1	11.8 ± 11.3	0.125 ^a^
Peak Angular Velocity [°/s]	161.6 ± 39.8	184.0 ± 37.7	0.020 ^a^
% Variation Peak Angular Velocity	23.6 ± 10.6	17.8 ± 10.4	0.271 ^a^

^a^ Parametric comparison corrected for age, sex, and UPDRS III; (TUG Timed up and go test).

## Data Availability

The data presented in this study are available on request from the corresponding author.

## Supplementary Materials

The following supporting information can be downloaded at: https://www.mdpi.com/article/10.3390/s26092594/s1, File S1: MRIq–Motor Reserve Index Questionnaire. Table S1: Turning characteristics of the subjects.
