# Supplementary material for: Wearable Inertial Sensor Analysis of Turning Performance Reveals Motor Reserve Effects in Drug-Naïve Parkinson’s Disease
Source: Sensors (Basel). 2026 Apr 22;26(9):2594. doi: 10.3390/s26092594 (PMC13165994; doi:10.3390/s26092594)
Supplement: Supplementary file 1 [file sensors-26-02594-s001.zip › sensors-4226163-File S1.pdf]

MRIq – Motor Reserve Index Questionnaire

Last name: ____________________ First name: ____________________

Date of birth: __________ Place of birth: __________ Age: _______

Employed: YES ■ NO ■

If NO, indicate retirement age: _______

Household activities

1. For how many years have you performed LIGHT household activities (e.g. sweeping, washing dishes)? ____

2. For how many years have you performed MODERATE household activities (e.g. ironing, cleaning floors, washing clothes by hand)? ____

3. For how many years have you performed HEAVY household activities (e.g. carrying firewood, washing windows, painting)? ____

Walking / mobility

1. For how many years have you climbed FLIGHTS OF STAIRS? ____

2. For how many years have you walked SHORT DISTANCES (e.g. going to buy bread, taking the dog out)? ____

3. For how many years have you walked LONG DISTANCES (>1 km)? ____

Leisure activities

1. For how many years have you engaged in leisure activities WHILE SITTING (e.g. DIY, small repairs, knitting)? ____

2. For how many years have you engaged in leisure activities WHILE STANDING (e.g. vegetable garden, gardening, hunting)? ____

Physical exercise

1. For how many years have you practiced LIGHT/MODERATE sports (e.g. bowls, dancing, jogging)? ____

2. For how many years have you practiced HEAVY/COMPETITIVE sports (e.g. running, cycling, tennis, soccer, basketball, volleyball)? ____

Caring activities

1. For how many years have you taken care of young children (e.g. grandchildren or children)? ____

2. For how many years have you taken care of an elderly or ill family member (spouse, parent)? ____

Work-related activities

1. For how many years did you perform a job involving LIGHT-intensity activities? ____

2. For how many years did you perform a job involving MODERATE-intensity activities leading to increased heart rate or breathing (e.g. brisk walking)? ____

3. For how many years did you perform a job involving HIGH-intensity activities leading to increased heart rate or breathing (e.g. moving heavy loads)? ____

4. For how many years did you perform a job requiring standing for more than 1 hour? ____

5. For how many years did you perform a job involving walking? ____
