# Supplementary material for: Wearable Inertial Sensor Analysis of Turning Performance Reveals Motor Reserve Effects in Drug-Naïve Parkinson’s Disease
Source: Sensors (Basel). 2026 Apr 22;26(9):2594. doi: 10.3390/s26092594 (PMC13165994; doi:10.3390/s26092594)
Supplement: Supplementary file 1 [file sensors-26-02594-s001.zip › sensors-4226163-Table S1.pdf]

Supplementary Table S1. Turning characteristics of the subjects

TURNING PARAMETERS	HC	PD	p-value
Participants (n)	40	45	
	TUG Self Selected Speed		
TUG Duration [s]	11.3±2.1	17.8±7.8	0.001[a]
Duration of turns [s]	2.3±0.4	3.0±0.7	0.003[a]
Mean Angular Velocity [°/s]	82.0±14.1	60.9±12.8	0.001[a]
Peak Angular Velocity [°/s]	194.3±34.2	136.6±26.4	0.001[a]
	TUG Fast Speed		
TUG Duration [s]	8.9±1.8	13.3±4.2	0.001[a]
% Variation Tug Duration	-21.8±8.8	-26.1±9.8	0.042[b]
Duration of turns [s]	1.9±0.3	2.6±0.6	0.014[a]
% Variation Duration of Turns	-16.6±12.6	-18.0±16.2	0.315[b]
Mean Angular Velocity [°/s]	98.2±18.1	73.3±15.2	0.001[a]
% Variation Mean Angular Velocity	20.9±16.4	11.8±11.3	0.045[b]
Peak Angular Velocity [°/s]	235.4±39.8	172.0±38.4	0.001[a]
% Variation Peak Angular Velocity	22.6±13.2	20.8±10.4	0.471[b]

a Parametric comparison corrected for age, sex

b Non-parametric comparison corrected for age, sex

(TUG Timed up and go test)
